# Supplementary material for: Tubulin-Dependent Transport of Connexin-36 Potentiates the Size and Strength of Electrical Synapses
Source: Cells. 2019 Sep 25;8(10):1146. doi: 10.3390/cells8101146 (PMC6829524; doi:10.3390/cells8101146)
Supplement: Supplementary file 1 [file cells-08-01146-s001.pdf]

cells

MDPI

Supplementary Materials

```
rCx36        296 VLNLAELNHLGWRKIKLAVRGAQAKRKSVYEI-RNKDLPRVSVP-NFGR----------- 312
rCx26        204 LLNITELCYLFIRYCSGKSKRPV------------------------------------- 226
rCx30        204 LLNVAELCYLLLKLCFRRSKRTQA----QRNH--PNHALKESKQNEMN-----------E 246
rCx31        200 ILTICEICYLIFHRIMRGLSKDKSTKSISSPK----S---SSRASTCR---------CHH 243
rCx31.1      197 LLNLVELLYLVIKRCSECAPAKRPPTAHAKND--PNWANPSSKEKDFL---------SSD 245
rCx32        203 ILNVAEVVYLIIRACARRAQRRSNPPSRKGSG-FGHRLSPEYKQNEIN---------K-- 250
rCx37        222 VLNLLELVHLLCRCVSREIKARRDHDTRPAQG----SASDPYPEQV-------------- 263
rCx40        219 FLSLAELYHLGWKKIRQRLAKSRQGDKH-------QLLGPST--------SLVQ-----G 258
rCx43        222 ALNIIELFYVFFKGVKDRVKGRSDPYHATTGP--------LSPSKDCG---------SPK 264
rCx46        222 VLNMLEIYHLGWKKLKQGVTNHFNPDA--SEV-RHKPLDPLSEAANSGPPSVSI-----G 273
rCx47        279 LLNLCEMAHLGLGSAQDAVRGRRGASAAG--------PGPAPRPPPCAFPAAAA-----G 325
rCx50        225 FLNIMEMSHLGMKGIRSAFKRPAEQPLGEIAEKSLHSIAVSSIQKAKGYQLLEEEKIVSH 284
                  *.: *: ::
```

Figure S1. Carboxyl terminal sequence alignment across connexin isoforms.

Sequence alignment of the carboxyl terminal of Cx36 to several connexin isoforms. The putative tubulin binding motif of Cx36, shaded in blue. Conserved residues within the Cx36 CT are indicated by the asterisks. Both CaM and CaMKII binding sites appeared to share some overlap with the tubulin-binding motif in the Cx36 protein, however, this signature motif is absent in the other isoforms. The CaMKII binding motif is outlined in blue and the CaM motif in red.

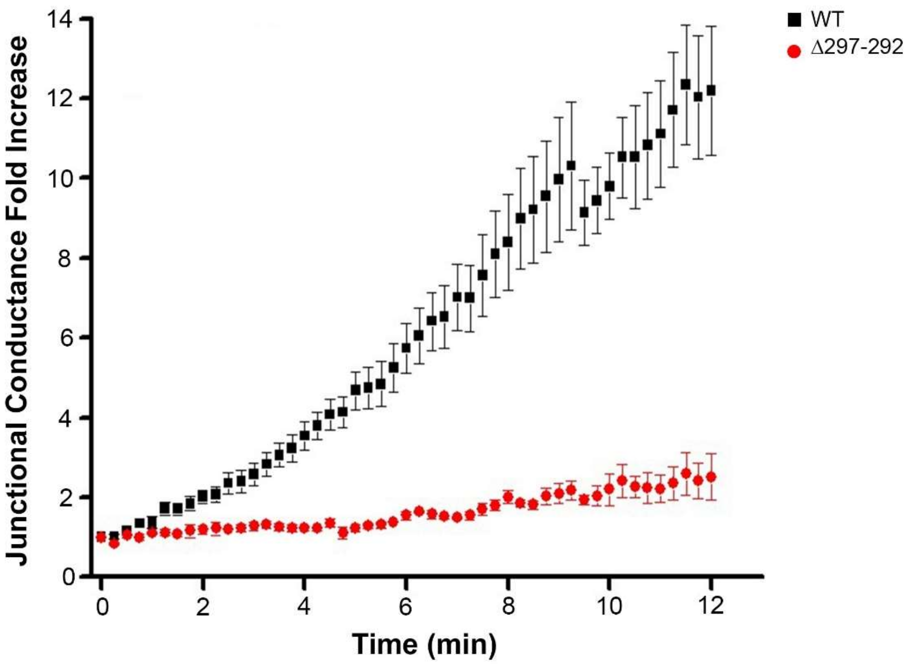

Figure S2. Run-up is abolished in the absence of the tubulin binding motif.

Electrical coupling was measured using the dual whole cell patch clamp technique in Neuro-2a cells pairs transiently transfected to express Cx36-EGFP. Expression of Cx36Δ279-292-EGFP abolished the run-up suggesting that the identified CT binding motif potentiates electrical plasticity.

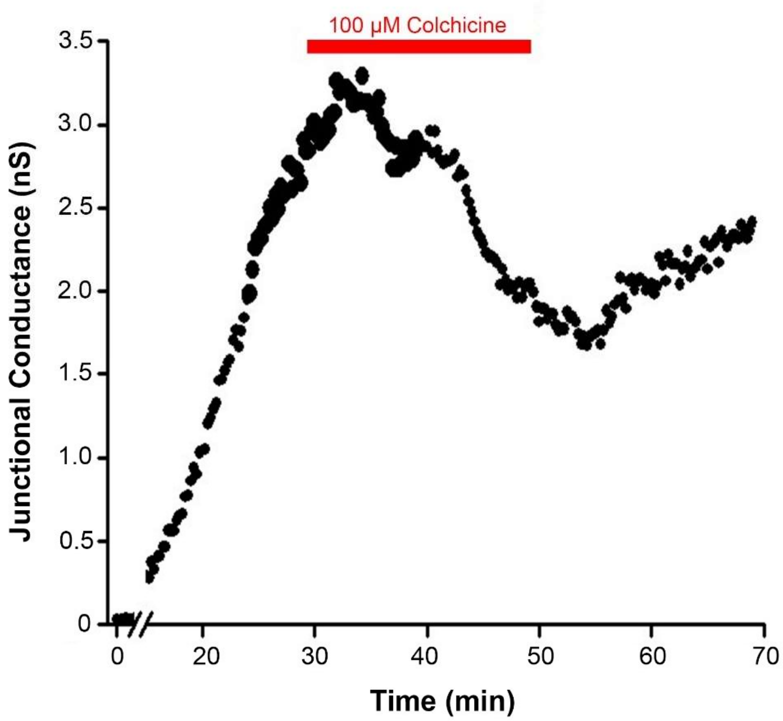

Figure S3. Run-up is reversibly abolished with colchicine application.

A 20 min application of Colchicine was potent enough to impair Cx36-mediated GJ conductance; this effect was reversible upon removal of Colchicine-supplemented bathing solution.

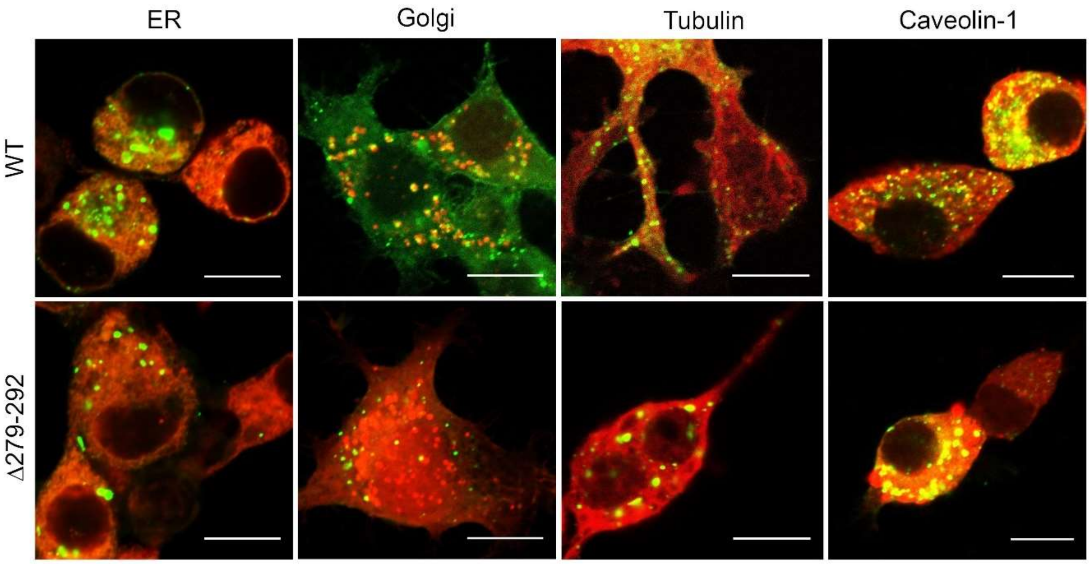

Figure S4. Colocalization image examples of Cx36 to various organelles.

Co-localization was determined by Mander's overlap coefficient. Cells were co-transfected with wildtype or mutant (Δ279-292) Cx36-EGFP and mCherry-tagged tubulin or the DsRed-monomeric protein tagged with the ER retention signal KDEL, amino acids 1-60 of human galactosyltransferase, or caveolin-1.

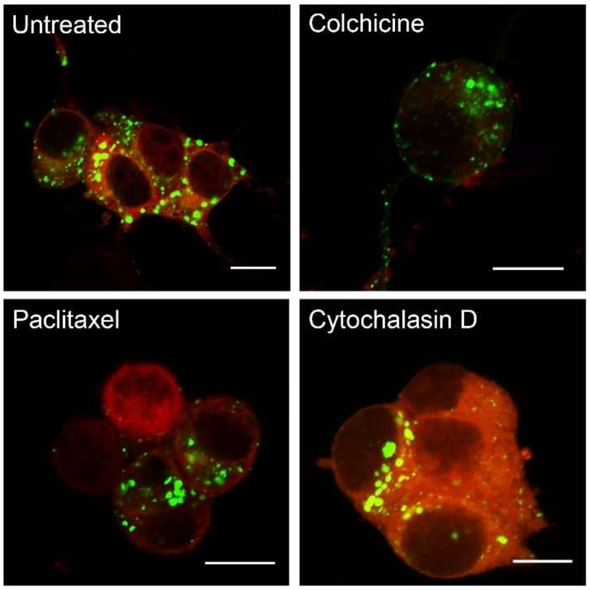

Figure S5. Colocalization image examples of Cx36 to the tubulin-cytoskeleton under pharmacological manipulation.

Co-localization was determined by Mander's overlap coefficient. Cells were co-transfected with wildtype Cx36-EGFP and mCherry-tagged tubulin and subsequently treated with colchicine, paclitaxel or cytochalasin D. Untreated cells were reserved as a control.

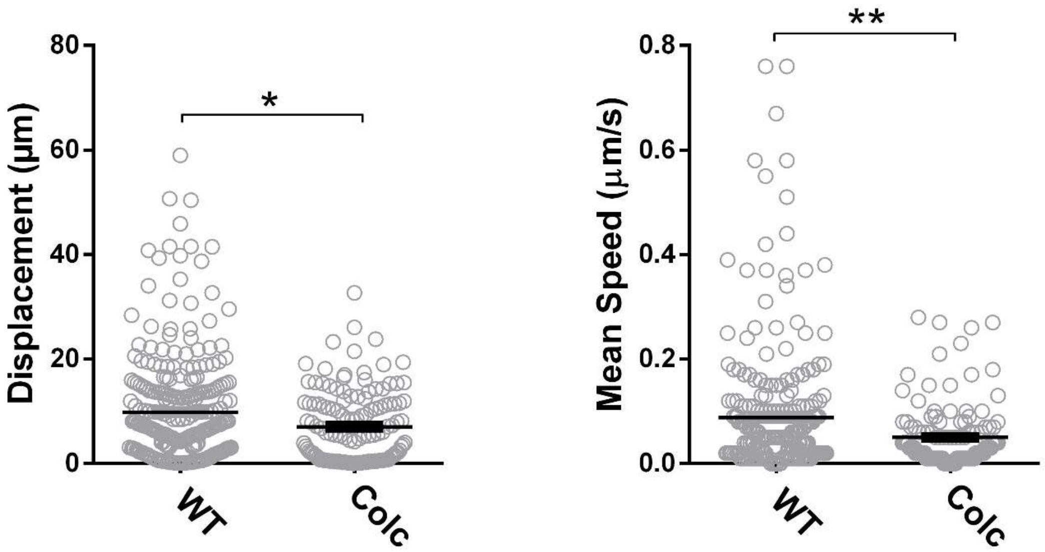

Figure S6. TIRF analysis of transport in single transfected N2a cells.

N2a cells were transfected to express wild type Cx36-EGFP and subsequently treated with colchicine to inhibit tubulin polymerization. Untreated cells served as the control.

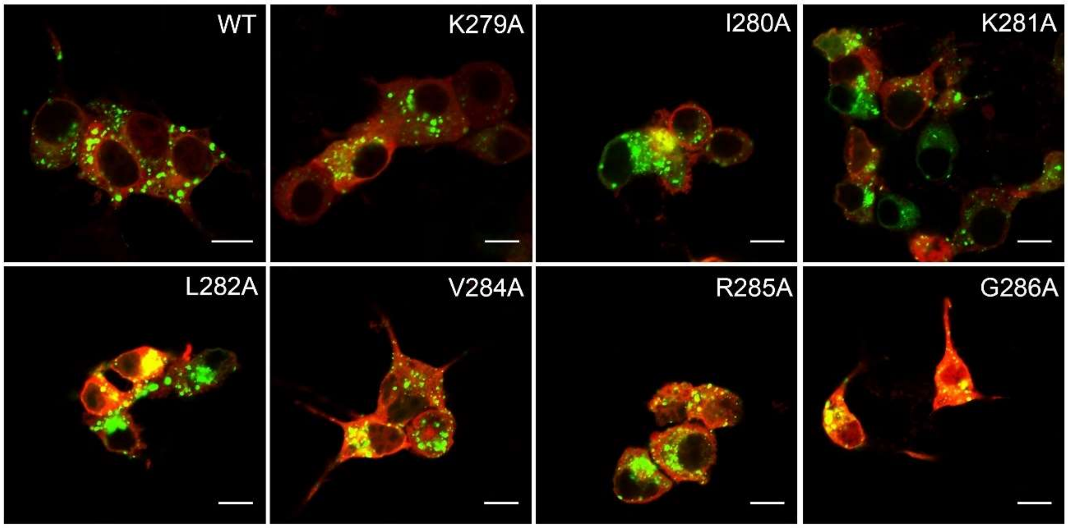

Figure S7. Colocalization image examples of various Cx36 mutants to the tubulin-cytoskeleton.

Co-localization was determined by Mander's overlap coefficient. Cells were co-transfected with wildtype or Cx36-EGFP and mCherry-tagged tubulin.
